# Staging quality is related to the survival of women with endometrial cancer: a Scottish population based study.Deficient surgical staging and omission of adjuvant radiotherapy is associated with poorer survival of women diagnosed with endometrial cancer in Scotland during 1996 and 1997

**DOI:** 10.1038/sj.bjc.6600358

**Published:** 2002-06-17

**Authors:** S C Crawford, L De Caestecker, C R Gillis, D Hole, J A Davis, G Penney, N A Siddiqui

**Affiliations:** Department of Gynaecological Oncology, Stobhill Hospital Glasgow G21 3UW, UK; Department of Public Health, Greater Glasgow Health Board, Dalian House, 350 St Vincent Street, Glasgow G3 8YT, UK; West of Scotland Cancer Surveillance Unit, University Department of Public Health, University of Glasgow G12 8RZ, UK; Scottish Programme for Clinical Effectiveness in Reproductive Health, Aberdeen Maternity Hospital, Aberdeen AB25 2ZL, UK

**Keywords:** endometrial cancer, survival analysis, staging, population study, Scotland

## Abstract

The association between treatment variation and survival of women with endometrial cancer was investigated. A retrospective cohort based upon the complete Scottish population registered on in-patient and day-case hospital discharge data (Scottish Morbidity Record-1) and cancer registration (Scottish Morbidity Record-6) coded C54 and C55 in ICD10, between 1st January 1996 to 31st December 1997 were analysed. Seven hundred and three patients who underwent surgical treatment out of 781 patients that were diagnosed with endometrial cancer in Scotland during 1996 and 1997. The overall quality of surgical staging was poor. The quality of staging was related to both the year that the surgeon passed the Member of the Royal College of Obstetricians and Gynaecologists examination and also to ‘specialist’ status but was not related to surgeon caseload. Two clinically important prognostic factors were found to be associated with survival; whether the International Federation of Obstetrics and Gynaecology stage was documented, RHR=2.0 (95% CI=1.3 to 3.1) and also to the use of adjuvant radiotherapy, RHR=2.2 (95% CI=1.5 to 3.5). The associations with survival were strongest in patients with advanced disease, International Federation of Obstetrics and Gynaecology stages 1C through to stage 3. Deficiencies in staging and variations in the use of adjuvant radiotherapy represent a possible source of avoidable mortality in patients with endometrial cancer. Consequently, there should be a greater emphasis on improving the overall quality of surgical staging in endometrial cancer.

*British Journal of Cancer* (2002) **86**, 1837–1842. doi:10.1038/sj.bjc.6600358 www.bjcancer.com

© 2002 Cancer Research UK

## 

Endometrial cancer is the second most common gynaecological cancer in the UK (Coleman *et al*, 1999) with approximately 400 cases diagnosed annually in Scotland. International comparisons show that survival in the UK, and in particular Scotland, is poor compared to other European countries (Gatta *et al*, 1998) but this data, from cancer registries, does not reveal the reasons for poor survival. There is little published in the literature describing variations in the management of this disease. An audit in south-east England found that inappropriate management was related to poorer survival outcome (Tilling *et al*, 1998). Endometrial cancer, unlike other gynaecological cancers, has traditionally been regarded as easy to treat (Lawton, 1997) nevertheless 25% of women will die of recurrence within 5 years of diagnosis (ISD, 2000).

The overall aim of this study was to improve the understanding of variations in survival of women with endometrial cancer in Scotland. Specifically the objectives of the Scottish endometrial cancer study were to describe current practice, to investigate the consistency of staging and to relate these to survival outcomes. Some of this other information will be reported elsewhere. This study describes the relationship between variations in the adequacy of surgical staging and the use of adjuvant radiotherapy with patient survival. This is an important issue as a greater emphasis in the quality of staging represents a ‘correctable’ aspect of clinical management that may lead to improved patient survival. This is particularly relevant, as national cancer plans drawn up for England (Department of Health, 1999, 2000), and in preparation for Scotland have been stimulated in part by the unfavourable comparisons between the UK and Europe.

## METHODS

The study was a retrospective case note review of all women with endometrial carcinoma who were resident in Scotland with a diagnosis first made between 1 January 1996 and 31 December 1997, the latest years for which complete data are currently available. Cases of endometrial carcinoma were identified from the Scottish Morbidity Record (SMR-1; in-patient and day case hospital discharge data). Cases were defined as patients who were coded as C54 and C55 in the 10th revision of the International Classification of Disease (ICD10). Prior to March 1996, the equivalent codes in ICD9 were used. At the end of the study, Cancer Registration (SMR-6) and SMR-1 data sets were linked to ensure completeness and any additional records were reviewed.

Prior to this study, a pilot study examining case records was conducted at a teaching hospital and at a district general hospital. This allowed the generation of hypotheses and informed the choice of variables to be collected. Data was collected from hospital medical records on diagnosis and staging, surgical treatment and adjuvant radiotherapy. Two experienced clinical data abstractors recorded data according to definitions pre-defined by the study committee. The study was conducted under the auspices of the Scottish Programme for Clinical effectiveness in Reproductive Health (SPCERH) and permissions were sought from MREC, the privacy committee of the Information and Statistics Division, hospital trusts and all consultant gynaecologists in Scotland. Data was collected from both the hospital of the definitive operation and radiotherapy centres. Pathology reports were reviewed by one of the investigators with experience in gynaecological oncology (SCC) who assigned a ‘retrospectively derived’ FIGO (International Federation of Obstetrics and Gynaecology) stage to every case. This was based upon the best available information from the clinical and pathology reports using the published FIGO staging nomenclature (Shepherd, 1989). If the cytology result was unavailable the result was assumed to be negative for the purpose of allocating a FIGO stage. Cases were defined as ‘unstageable’ if there was no operation, there was insufficient histological information or if there were synchronous tumours present. The data abstractors cross-checked 1 in 50 (24) abstracted records for accuracy. Data was entered into an Access-97 database (Microsoft Inc., 1997) and statistical analysis was carried out in SPSSv9.0 for Windows (SPSS Inc., 2000).

Patients were grouped into four categories on the basis of their retrospective FIGO stage. These groups represented the likelihood of metastatic spread and thus the use of adjuvant radiotherapy: ‘low risk’ of metastatic spread (FIGO stages 1AG1 and 1BG1), ‘intermediate risk’ (FIGO stages 1AG2/G3, 1BG2/G3, 1CG1/G2), ‘high risk’ (FIGO stages 1CG3 and stages 2/3/4) and ‘unstageable’. The ‘intermediate’ risk group represents the study group of a recent randomised trial of post-operative radiotherapy in endometrial cancer (Creutzberg *et al*, 2000).

Survival data were obtained by computerised probability matching to the Registrar General's death records (Kendrick and Clarke, 1993). The date of censoring was 31st March 2000. We found 59 cases of proven endometrial cancer that were not linkable to the Registrar General's death records. These cases were included in the analysis. In these cases the date of censoring was defined as the date of data abstraction from the case record if there was no indication of death. The rationale for this was that case records are usually ‘marked’ by medical record' staff when a patient becomes deceased. The Carstairs classification of socio-economic deprivation (Carstairs and Morris, 1991) was used to allocate patients to categories of socio-economic deprivation. The seven categories were aggregated to three categories (1 and 2, 3–5, 7 and 8) to facilitate analysis.

The results of the initial pilot audit indicated that staging was poorly performed. Two aspects of staging were examined, whether fluid was sent for cytological examination and whether the FIGO stage was calculated and documented in the medical record by either the surgeon or the pathologist. We used multiple logistic regression to explore four factors that were initially perceived to have a potential bearing on the quality of surgical staging. These were ‘specialist’-gynaecological surgeons; surgeon caseload, the date of postgraduate education (MRCOG pass date) and hospital volumes. For the purposes of this study a gynaecology cancer ‘specialist’ was defined as a gynaecologist performing radical surgery for cervical carcinoma in 1996/7. Prior to the study, it had been noted that some senior clinicians were less likely to perform staging as thoroughly as younger consultants. Until 1988, the staging of endometrial cancer was based on the results of a clinical examination under anaesthesia. The International Federation of Obstetrics and Gynaecology (FIGO) introduced a system in 1988 that involved combining information collected at the time of surgery with histological data from the subsequent pathology report. This change was first reported in the UK literature in 1989 (Shepherd, 1989). Each gynaecologist was categorised according to their year of passing the examination of Member of the Royal College of Obstetricians and Gynaecologists (MRCOG) (RCOG, 1997). In each case the senior surgeon present at operation was classified as obtaining MRCOG before or after 1989. This allowed gynaecologists to be categorised according to the FIGO staging system that they had been accustomed to during their early training. Surgeon caseload was represented by the number of cases of endometrial cancer that had been treated over the 2 years of the study cohort. Likewise the hospital volume represents the number of cases of endometrial cancer that were treated in 1996/7.

A number of possible factors that might be related to survival were explored.

Univariate analysis was used to relate each possible prognostic factor with survival using the Kaplan–Meier method. The Log rank statistic was used to compare individual survival curves. The primary end point in the survival analysis was death from any cause. This was used instead of the cancer specific cause of death due to acknowledged inaccuracies in death certificate information (Maudsley and Williams, 1993). In the final analysis a Cox proportional hazards model was used (Katz, 1999).

## RESULTS

One thousand and eighty five possible cases were identified from SMR-1 and a further 149 cases from SMR-6. Of these, 67 records could not be located and 299 cases were excluded of these 172 cases were diagnosed out with 1996/7 and 127 cases were tumours other than uterine cancer. A further 87 cases of uterine sarcoma were also excluded. Thus, 781 patients with endometrial carcinoma diagnosed in 1996/7 were available for analysis. Of these, 703 were initially treated by surgery and this is the group discussed in this study.

The FIGO stage was defined in the case record by the surgeon and/or pathologist in only 36.4% of cases despite the fact that extent of invasion and tumour grade was present in 88.6% of case records. Fluid was sent for cytological examination in only 46.6% of cases. The intra-peritoneal cytology rate can be validated. A 98% concurrence was found between the operation record and whether a cytology report was issued. This suggests that if this aspect of staging was performed then it was recorded in the operation record.

Table 1

**Table 1:**
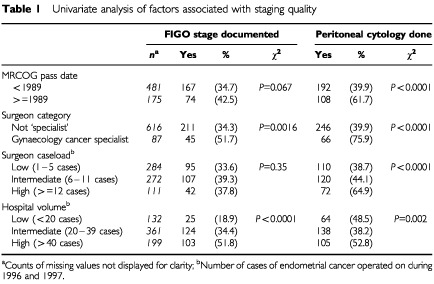
Univariate analysis of factors associated with staging quality

shows the surgeon and workload factors that were associated with more comprehensive staging. Table 2

**Table 2:**
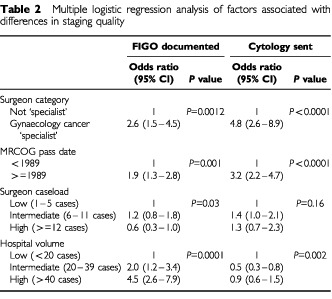
Multiple logistic regression analysis of factors associated with differences in staging quality

shows the results of the multiple logistic regression analysis that was used to identify the strengths of association between the factors. In each of the analyses there is an increased likelihood of more adequate staging by surgeons passing the MRCOG after 1989 or having ‘specialist’ status. No independent association was observed between surgeon caseload and staging quality despite the observation that ‘specialists’ had higher caseloads than non-specialists. Half (four) of the ‘specialists’ had a caseload greater than 12 cases during 1996/7 compared with only 3% of non-specialists. Indeed only one ‘specialist’ performed less than six cases compared with 65% of non-specialists. Hospital volume had an inconsistent association with staging quality. Higher volume hospitals were more likely to document the stage but were less likely to send material for cytological examination.

Survival data were obtained to 31st March 2000. The follow up for patients ranged from 2.25 to 4.25 years. This follow up time is sufficient to demonstrate that there are important differences in survival. At the date of censoring there had been 119 (17%) deaths in those patients who had had surgical treatment.

Table 3

**Table 3:**
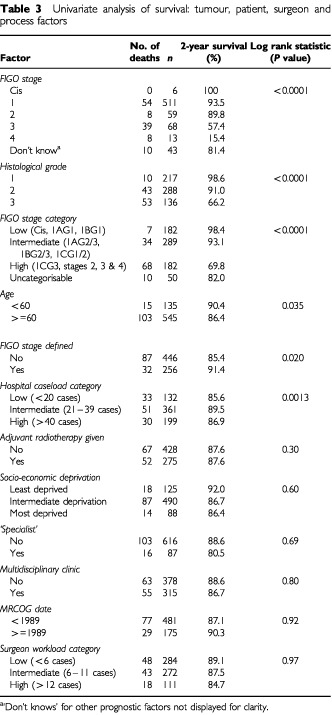
Univariate analysis of survival: tumour, patient, surgeon and process factors

is a univariate survival analysis showing the association of a number of variables with survival. These variables relate to the patient, their tumour, and the operating surgeon and other factors relating to processes of care. Six factors were found to be statistically significant: FIGO stage (*P*<0.0001) and tumour grade (*P*<0.0001), FIGO stage category (*P*<0.0001), hospital caseload category (*P*=0.0013), documentation of the FIGO stage (*P*=0.02) and the patient age category (*P*=0.035).

The strength of association of the various factors was modelled in a Cox's proportional hazards model. The results are shown in Table 4

**Table 4:**
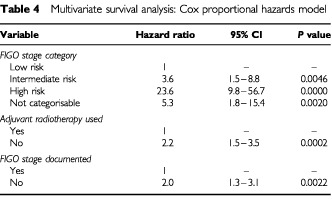
Multivariate survival analysis: Cox proportional hazards model

. The factors that were independently significant, after adjustment for the others, are the retrospectively assigned FIGO stage category (*P*<0.0001), whether adjuvant radiotherapy was used (*P*=0002) and whether the patient's FIGO stage was documented (*P*=0.0022).

‘Attendance at a multidisciplinary clinic’ was a statistically significant prognostic factor with a hazard ratio of 0.63 (95% CI: 0.42–0.94) when the model was run without ‘FIGO stage documented’ and ‘adjuvant radiotherapy used’.

**Figure 1:**
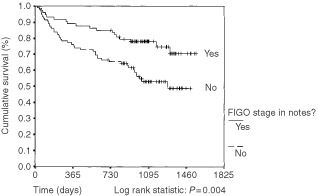
Kaplan–Meier survival curve: association of documentation of FIGO stage with survival: FIGO stage 1CG3, stage 2 and stage 3 disease only. FIGO stage documented in 73 patients *vs* not documented in 92 patients. Vertical bar represents censored cases.

**Figure 2:**
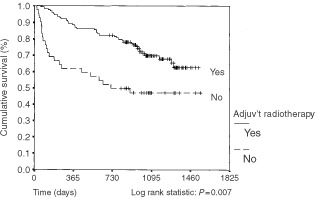
Kaplan–Meier survival curve: association of use of adjuvant radiotherapy with survival in patients with FIGO stage 1CG3, stage 2 and stage 3 disease only. One hundred and twenty-four patients received adjuvant radiotherapy *vs* 42 patients not receiving radiotherapy. Vertical bar represents censored cases.

More detailed analysis was performed to determine whether there were specific FIGO stages where staging and the use of adjuvant radiotherapy were associated with differences in survival. These associations were statistically significant in stages 1CG3 through to stage 3 only. Figure 1

shows the Kaplan–Meier survival curves for this subgroup. The two curves are significantly different, (log rank statistic: *P*=0.004). Similar analysis on this subgroup was performed looking at the effect of adjuvant radiotherapy on survival. The Kaplan–Meier survival curves are shown in Figure 2

. The survival curves are significantly different (log rank statistic: *P*=0.0007). We performed a Cross-tabulation analysis to examine the stage distribution between those patients where stage was or was not documented in the notes. The stage distribution between the two groups was not statistically different, χ^2^: *P*=0.21. Similarly the stage distribution between those patients receiving radiotherapy and not receiving radiotherapy was examined and no evidence of any difference was found, χ^2^: *P*=0.51. Thus it is likely that these are genuine differences in survival, representing the association between staging being documented and of adjuvant radiotherapy being used.

Table 5

**Table 5:**
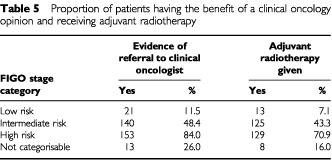
Proportion of patients having the benefit of a clinical oncology opinion and receiving adjuvant radiotherapy

shows the proportion of patients being referred to, or discussed with, a clinical oncologist and the proportion of patients in each FIGO stage category who received adjuvant radiotherapy.

## DISCUSSION

One of the strengths of this study is that the data relate to a well-defined national population with a robust mechanism for case identification and record retrieval. We also believe that the data quality is high. Cases were identified independently from two sources, SMR-1 and SMR-6, and over 95% of identified records were located and abstracted. The actual histo-pathology report was available in 96% of cases. The pathology report was missing from the case record in the remaining 4%, however the diagnosis was confirmed in correspondence between consultant and general practitioner.

A new finding was the association between the MRCOG pass date and the quality of staging. Taken together the ‘specialist effect’ and the ‘MRCOG date’ point towards knowledge being the common factor that results in improved staging quality. Younger gynaecologists may have more up to date knowledge of the current staging system and may be more aware of the benefits of staging. No independent association was found between surgeon caseload and staging quality despite the fact that 80% of cases were performed by gynaecologists operating on less than six cases a year. The association between hospital volume and staging quality is inconsistent. These findings support data from other studies where specialist surgeons were observed to stage more adequately (Kingsmore *et al*, 1998). Of note is the fact that basic staging procedures are not technically difficult. Sending intra-peritoneal washings for cytology requires no particular skill, only to remember that they should be taken and a belief that it is worthwhile. Likewise, recording the FIGO stage in the notes requires recognition of its importance, knowledge of the staging system and remembering to do it. ‘Specialists’ were more likely to document stage. These findings support the idea that part of the ‘specialist effect’ is a greater understanding of the reasons for the surgery performed.

The survival analysis demonstrated that the process of staging remained a statistically significant prognostic factor even after adjusting for other known factors including the use of adjuvant radiotherapy. This confirms and emphasises its importance. It is not proposed that this process itself confers a direct surgical benefit. This point is important since advanced staging procedures such as pelvic lymphadenectomy and para-aortic lymphadenectomy, more commonly performed in other countries such as the United States, have been argued as surgically therapeutic in their own right (Orr, 1998). These more aggressive staging procedures were uncommonly performed in this Scottish cohort (4% patients had pelvic lymphadenectomy and only 0.57% had para-aortic lymphadenectomy). It is more likely that staging is a proxy marker of overall quality in the patient management process.

At the time of defining the stage, all relevant details pertaining to the disease are brought together. This collation and standardisation probably facilitates decision-making. This is important where a patient requires referral to another clinician for further management. This situation is common in the management of many cancers. The association of the multidisciplinary clinic to survival has been previously shown for ovarian cancer (Junor *et al*, 1994). Attendance at a multidisciplinary clinic is statistically significant for patients in this study only when the variables, ‘staging documented’ and ‘use of adjuvant radiotherapy’ are removed from the multivariate analysis. This suggests that in this cohort at least, part of the ‘multidisciplinary effect’ may be related to the adequacy of staging and the concomitant use of adjuvant radiotherapy in ‘high risk’ patients. This means that the multidisciplinary clinic might contribute to patient survival by providing a mechanism to ensure that those patients who need adjuvant treatments actually receive them. This point is emphasised by the data in Table 5.

The use of adjuvant radiotherapy was a significant prognostic factor too. The effect of adjuvant radiotherapy was particularly important in more advanced disease (Figure 2). Adjuvant radiotherapy is known to reduce local recurrence, however its effect on actual survival outcome is less certain (Lawton, 1997). These results suggest that adjuvant radiotherapy is associated with improved survival in patients with advanced disease.

‘Specialist’ status and the year of the surgeons' MRCOG examination had no independent association with survival in this multivariate model. The lack of an independent ‘specialist effect’ suggests that it is more important what is done than who does it. However, ‘specialists’ operated on only 87 cases (12%) and the study may have been underpowered to determine the true strength of this relationship. It is acknowledged that there may be some disagreement with our definition of a ‘specialist’, however this was a pragmatic definition which aimed to define those clinicians who both had a ‘declared’ interest in gynaecological cancer and who had the surgical capability to manage endometrial cancer.

Further analysis was performed to see whether there were specific groups of patients who might have benefited from better quality staging or adjuvant radiotherapy. The prognostic benefit of staging and of radiotherapy was limited to FIGO stages 1CG3, stage 2 and stage 3. A proportion of those patients (25.3%) did not receive adjuvant radiotherapy. This probably represents genuine under-treatment of disease in this group. Moreover, many of these patients had no stage documented, despite the fact that the information to do so was contained within the patient's case notes.

## CONCLUSIONS

This study shows that the overall quality of staging was poorly performed and that adjuvant radiotherapy was inconsistently used particularly in more advanced tumours. Staging, as a process, is a prognostic factor particularly in patients with more advanced cancer. It is likely that the main benefit of staging is to provide key information required for subsequent clinical management decisions, particularly within the multidisciplinary context. One of the most important decisions is whether the patient should receive any further treatment. These results support a greater emphasis towards improving the overall quality of surgical staging. In particular there needs to be an improvement in the understanding of the purpose of surgery, not just to remove the cancer, but also to provide the information required for subsequent management decisions. This is especially important in Scotland where endometrial cancer continues to be managed by general gynaecologists rather than specialist gynaecological oncologists. These results support the strategies being developed for the management of endometrial cancer in England.
